# Neu1 sialidase and matrix metalloproteinase-9 cross-talk in alliance with insulin receptors is an essential molecular signaling platform for insulin-induced receptor activation

**DOI:** 10.1186/1471-2164-15-S2-P22

**Published:** 2014-04-02

**Authors:** Farah G Alghamdi, Myron Szewczuk

**Affiliations:** 1 Department of Biomedical & Molecular Sciences, Queen’s University, Kingston, Ontario, Canada

## Background

Molecular-targeting therapeutics directed towards growth factor receptors have become promising interventions in cancer. They include the family of mammalian receptor tyrosine kinases such as epidermal growth factor, nerve growth factor TrkA and insulin. In particular, the insulin receptor (IR) is one of the most well-known members of the RTK family of receptors playing a role in cancer. IRs are covalently-linked heterodimers of αβ subunits on the cell membrane in the absence of insulin binding. The IR signalling pathways are initially triggered by insulin binding to the extracellular portion of α subunits followed by the interaction of β subunits and ATP. The parameter(s) controlling insulin-induced conformational change of the receptor and activation remains unknown.

## Materials and methods

Three different cell lines were used: HTC-IR (rat hepatoma cells overexpressing human insulin receptor), HTC-WT and MiaPaCa-2 (human pancreatic carcinoma) cells. Several methods were used through the project such as; Sialidase assay, immunocytochemistry, colocalization, Western Blot, Co-Immunoprecipitation.

## Results

Here, we report a membrane receptor signalling platform initiated by insulin binding to its receptor to induce Neu1 in live HTC-IR, HTC-WT and MiaPaCa-2 cell. Tamiflu, galardin and piperazine (broad range MMP inhibitors), MMP9 inhibitor and anti-Neu1 antibody blocked Neu1 activity associated with insulin stimulated live cells. Tamiflu blocked insulin induced insulin receptor substrate-1 phosphorylation (phospho-IRS-1). Microscopy colocalization and Co-IP analyses reveal that Neu1 and MMP-9 form a complex with naïve and insulin-treated receptors.

## Conclusions

These findings uncover a molecular organizational signalling platform of a novel Neu1 and MMP-9 cross-talk in alliance with insulin receptors. It proposes that insulin binding to the receptor induces MMP9 to activate Neu1 which hydrolyzes α-2,3 Sialic acid in removing steric hindrance to generate a functional receptor. The results predict a prerequisite desialylation process by activated Neu1 enabling the removal of steric hindrance to receptor association. Neu1 is a promising novel cancer-targeting enzyme which is unaffected by most activating mutations in the RTK gene arsenal in cancer cells. A complete understanding of IR structure, activation and the role of Sialic acid in the signaling pathways may provide a therapeutic strategy in the prevention of different diseases such as diabetes mellitus and cancer.
